# Analysis of tumour-infiltrating lymphocytes reveals two new biologically different subgroups of breast ductal carcinoma in situ

**DOI:** 10.1186/s12885-018-4013-6

**Published:** 2018-02-03

**Authors:** Marie Beguinot, Marie-Melanie Dauplat, Fabrice Kwiatkowski, Guillaume Lebouedec, Lucie Tixier, Christophe Pomel, Frederique Penault-Llorca, Nina Radosevic-Robin

**Affiliations:** 1 Department of Surgical Oncology, Jean Perrin Comprehensive Cancer Centre, 58 rue Montalembert, 63011 Clermont-Ferrand, France; 2 Department of Surgical Pathology and Biopathology, Jean Perrin Comprehensive Cancer Centre, 58 rue Montalembert, BP392, 63011 Clermont-Ferrand, France; 3 Master Program « Biology & Health », University Paris-East Val-de-Marne (UPEC), 61 avenue du General de Gaulle, 94010 Creteil, France; 4 Department of Clinical Research, Jean Perrin Comprehensive Cancer Centre, 58 rue Montalembert, 63011 Clermont-Ferrand, France; 5 University Clermont Auvergne, INSERM U1240, Jean Perrin Comprehensive Cancer Centre, 58 rue Montalembert, 63011 Clermont-Ferrand, France; 6 Present Address: Department of Pathology, Paoli-Calmettes Comprehensive Cancer Centre, 232 boulevard Sainte-Marguerite, 13009 Marseilles, France

**Keywords:** breast, cancer, ductal, in situ, microinvasive, lymphocytes

## Abstract

**Background:**

Tumour-infiltrating lymphocytes (TILs) have been demonstrated to significantly influence prognosis and response to therapy of invasive breast cancer (IBC). Thus, it has been suggested that TIL density or/and immunophenotype could serve as biomarkers for selection of IBC patients for immunotherapy. However, much less is known about significance of TILs in breast ductal carcinoma in situ (DCIS).

**Methods:**

We retrospectively investigated TIL density and immunophenotype in 96 pure DCIS and 35 microinvasive carcinomas (miCa). TIL density was assessed on H&E-stained breast biopsy sections as the percentage of tumour stromal area occupied by TILs, and classified into 4 grades: 0 (0%–9%), 1 (10–29%), 2 (30–49%) and 3 (50%–100%). TIL immunophenotype was assessed by immunohistochemistry for CD8, CD4, FoxP3, CD38 or CD20.

**Results:**

Compared to pure DCIS, miCa contained significantly more cases with TIL density grade 3 (*p* = 0.028). Concordantly, CD8+, CD4+ and CD38+ cells were more numerous in miCa than in pure DCIS. In the pure DCIS subgroup with TIL density grades 2 and 3, all TIL subpopulations were more numerous than in the pure DCIS with TIL density grades 0 and 1, however the ratio between T-lymphocytes (CD8+ and CD4+) and B-lymphocytes (CD20+) was significantly lower (*p* = 0.029). On the other side, this ratio was significantly higher in miCa, in comparison with pure DCIS having TIL density grades 2 and 3 (*p* = 0.017). By cluster analysis of tumour cell pathobiological features we demonstrated similarity between miCa and the pure DCIS with TIL density grades 2 and 3. The only significant difference between those two categories was in the ratio of T- to B-TILs, higher in miCa.

**Conclusion:**

Results indicate that TIL density level can distinguish 2 biologically different DCIS subgroups, one of which (DCIS with ≥30% TILs, the TIL-rich DCIS) is like miCa. Similarity of TIL-rich pure DCIS and miCa as well as the role of B-lymphocytes in DCIS invasiveness are worth further investigating with regards to the potential development of immunotherapy-based prevention of DCIS progression.

**Electronic supplementary material:**

The online version of this article (10.1186/s12885-018-4013-6) contains supplementary material, which is available to authorized users.

## Background

Ductal carcinoma in situ (DCIS) now accounts for 20–25% of all female breast cancers (BCs) in countries with active mammographic screening [1]. Similarly to invasive breast cancer (IBC), DCIS displays significant heterogeneity at the clinical, morphological, molecular and prognostic level [2]. However, clinical management of DCIS patients (pts) is still quite uniform, with many issues of debate, particularly with regards to over- or undertreatment [3]. This situation is caused by a lack of reliable predictors of DCIS progression [4] and/or of molecular targets which could be therapeutically modulated to prevent occurrence of the invasive disease.

Impressive therapeutic results obtained in the recent years by modifiers of the immune response to cancer have initiated movements calling for intense investigation of the immune microenvironment of preinvasive malignant lesions [5]. It has been hypothesized that immunotherapies could prevent progression of the cancers in situ and even induce their rejection. Such a treatment would be particularly appealing to the patients with breast premalignant lesions, as the successful immunotherapies could reduce the rate of extensive surgeries and thus significantly improve patients’ quality of life.

Numerous studies have demonstrated important impact of tumour-infiltrating lymphocytes (TILs) on the natural or therapeutically-modified evolution of IBC [6]. On that basis, it has been proposed that TIL characteristics, like their density or immunophenotype, could help selecting IBC pts. for immunotherapy clinical trials [6, 7]. However, knowledge on TILs’ role in DCIS is still limited. Therefore, in the study presented here, we investigated the characteristics of stromal TILs in a larger series of DCIS pts. We demonstrate that histological assessment of TILs can help recognizing a subcategory of pure DCIS that is biologically very close to microinvasive carcinoma (miCa). The only difference between those two categories lies in the composition of TILs, so that observation might provide clues for better understanding and prevention of DCIS invasion.

## Methods

### Patients

Female pts., aged ≥18, with a unilateral breast DCIS, treated at the Jean Perrin Comprehensive Cancer Centre between 2001 and 2005, were retrospectively selected. The pts. with family history of BC, germline *BRCA1* mutations, with incomplete clinical annotations and/or lost from follow-up were excluded. Diagnosis of DCIS was initially established on breast biopsy and confirmed on the corresponding surgical specimen. The DCIS without any invasion were designated as pure DCIS (DCIS), whereas the microinvasive carcinoma (miCa) category comprised the in situ lesions with an invasive component of 1 mm or less in the greatest dimension [8]. The cases diagnosed as pure DCIS on biopsy but as miCa on surgical specimen were excluded. The final cohort consisted of 131 pts.

Median patient age was 56 [36–84] years. All the pts. had mastectomy or breast conservative surgery. After surgery, 92 pts. had adjuvant treatment whereas 39 pts. were only observed.

The median follow-up time was 144 [115–173] months. Eighteen pts. (14%) experienced recurrences: 7 non-invasive and 11 invasive. Two pts. developed distant metastases (one of them had also a locoregional recurrence). Among the locoregional recurrences 7 were non-invasive, 10 invasive, 10 ipsilateral, 6 contralateral, 1 bilateral. Details on patient characteristics are presented in Table 1.

**Table 1:** Patient characteristics

	all pts. (*n* = 131)	DCIS pts. (*n* = 96)	miCa pts. (*n* = 35)	*p* value(*)
mean age [range]	56 [36–84]	56 [36–84]	54 [38–78]	NS
initial management				
lumpectomy	89 (68%)	68 (71%)	21 (60%)	NS
mastectomy	42 (32%)	28 (29%)	14 (40%)	
adjuvant treatment				
radiotherapy	89 (68%)	68 (71%)	21 (60%)	NS
endocrine treatment	10 (8%)	1 (1.0%)	9 (26%)	< 0.0001
cytotoxic treatment	2 (2%)	0 (0.0%)	2 (6%)	0.03
observation	39 (30%)	27 (28%)	12 (34%)	NS
trastuzumab	1 (1%)	0 (0%)	1 (3%)	NS
recurrences	18 (14%)	14 (14%)	4 (11%)	NS
DFS at 5 years	94% (*n* = 124)	94% (*n* = 91)	94% (*n* = 34)	NS
DFS at 10 years	89% (*n* = 108)	88% (*n* = 78)	91% (*n* = 31)	

Abbreviations: *DCIS* pure DCIS, *miCa* microinvasive carcinoma, *DFS* disease-free survival, *NS* not significant, (*) characterizing the difference between DCIS and miCa

### Histological analysis and construction of tissue microarrays (TMAs)

Haematoxylin-eosin (H&E)-stained slides from formalin-fixed, paraffin-embedded breast biopsies and surgical specimens were reviewed by a senior breast pathologist. Lesion size, nuclear grade, mitotic index, architectural pattern and presence of necrosis and/or microcalcifications were recorded.

**Fig. 1:**
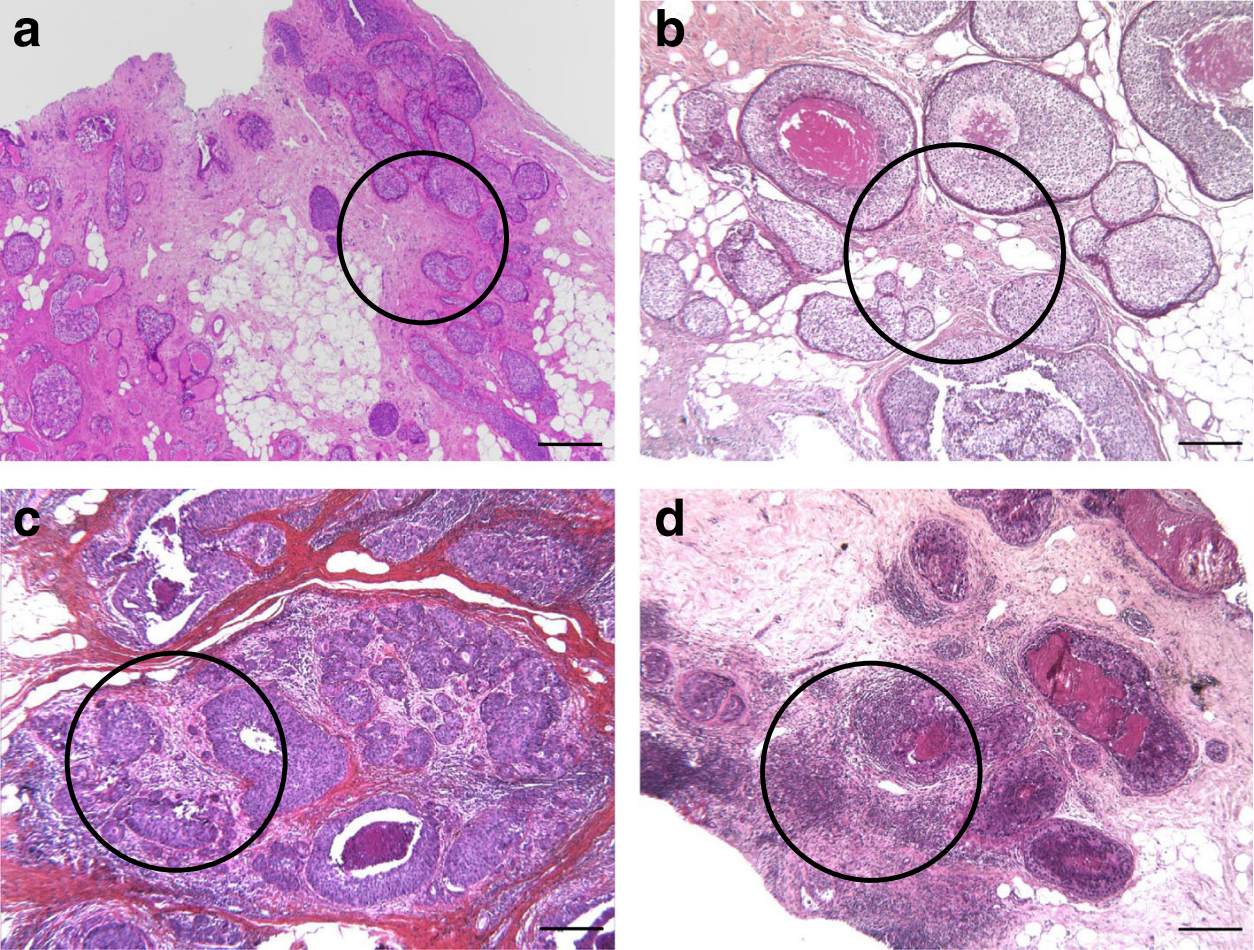
Examples of TIL density grades. **a**) a case representing TIL density grade 0 (TILs are estimated to occupy 2% of the stromal area). H&E, original magnification 4×, scale bar 250 μm; **b**) a case representing TIL density grade 1 (TILs are estimated to occupy 20% of the stromal area). H&E, original magnification 5×, scale bar 200 μm; **c**) a case representing TIL density grade 2 (TILs are estimated to occupy 40% of the stromal area). H&E, original magnification 5×, scale bar 200 μm; **d**) a case representing TIL density grade 3 (TILs are estimated to occupy 60% of the stromal area). H&E, original magnification 5×, scale bar 200 μm. The circles show the areas sampled for tissue microarray construction

Density of TILs was estimated semi-quantitatively as the percentage of tumour stromal area occupied by lymphocytes, lympho-plasmocytoid cells and plasmocytes, according to the recommendations for TIL density (TIL-d) assessment in IBC [9]. The area for analysis included the intra-tumour stroma plus the stromal area surrounding the CIS or miCa within one high-power field (× 40). TIL-d was classified into 4 grades: 0 (minimal, 0–9%), 1 (low, 10–29%), 2 (moderate, 30–49%) and 3 (high, 50–100%). Examples of the grades are represented on Fig. 1a-d, respectively.

TMAs were constructed by sampling 3 cylinders of 0.6 mm diameter from each tumour. The cylinders were taken from areas with the highest TIL-d, mostly from biopsies but also from surgical specimens, when biopsy was insufficient. At least 200 malignant cells of CIS must have been sampled for each case. In cases of miCa, one cylinder had to contain the microinvasive component and at least one cylinder the in situ component.

### Immunohistochemistry (IHC) and in situ hybridization (ISH)

IHC was performed on 3–4 μm thick TMA sections, using the procedures validated for diagnostics, in a fully automated system (Benchmark XT, Ventana). IHC detection of oestrogen receptor (ER), progesterone receptor (PR), HER2 and Ki67 was used for malignant cell characterization. The phenotype of TIL was assessed by IHC for CD8, CD4, FoxP3, CD20 and CD38. Details of the IHC procedures are presented in Table 2.

**Table 2:** Immunohistochemical procedures

Ag	Ab clone	Ab supplier	Ag retrieval	Ab dilution incubation time	Reveal system
ER	SP1	Ventana	CC2 36 min	ready-to-use, 32 min	ultraView DAB
PR	1E2	Ventana	CC2 36 min	ready-to-use, 20 min	ultraView DAB
HER2	4B5	Ventana	CC2 36 min	ready-to-use, 20 min	ultraView DAB
Ki67	SP6	Thermo Scientific	CC2 36 min	1/100, 60 min	ultraView DAB
CD8	SP16	Thermo Scientific	CC2 36 min	1/400, 60 min	ultraView DAB
CD4	SP35	Cell Marque	CC2 36 min	1/50, 60 min	ultraView DAB
FoxP3	2A11G9	Novus Biologicals	CC2 36 min	1/25, 60 min	ultraView Red
CD38	SP149	Cell Marque	CC2 36 min	1/50, 60 min	ultraView DAB
CD20	L26	Dako	CC2 36 min	1/100, 32 min	ultraView DAB

Abbreviations: *Ag* antigen, *Ab* antibody, *ER* oestrogen receptor, *PR* progesterone receptor, *CC1 or CC2* Cell Conditioning Buffer 1 or 2 (Ventana). All ultraView systems are from Ventana

IHC for ER and PR was interpreted according to Allred [10] and for HER2 according to the ASCO/CAP criteria [11]. *ERBB2* amplification was assessed by ISH in cases scored 2+ for HER2 expression by IHC. The fluorescent ISH for *ERBB2* was performed using Zyto*Light*® ERBB/CEN17 Dual Color Probe for in vitro diagnostics (ZytoVision GmbH, Bremerhaven, Germany), according to the manufacturer’s protocol. The ISH were interpreted using the ASCO/CAP criteria [11].

Molecular subtypes of DCIS were defined by applying the surrogate IHC classification recommended for IBC by the Saint Gallen International Expert Consensus [12]. For purpose of this study, luminal/HER2+ and HER2+/non-luminal subtypes were analysed as one category.

Number of TILs per mm2, labelled by each of CD8, CD4, FoxP3, CD20 or CD38, was determined as follows: percentage of TMA spot area occupied by stroma was determined visually on each spot and reported as increments of 10 (0–100%); the area of stroma available for TIL counting was obtained by multiplying the spot area (0,28 mm2) by the % of stroma; TILs were counted within the stroma; the value of TILs/mm2 was obtained by proportion calculation. Finally, mean TILs/mm2 count was derived from the counts obtained on the available spots for each case.

### Statistical analysis

The clinico-pathological data were recorded in a central database using SEM software [13]. The relationships between variables were analysed by Chi^2^ or Fisher’s exact test, Student t-test, Mann-Whitney U-test or Kruskal-Wallis H-test. When calculating ratios between counts, in cases in which the denominator was 0, that value was replaced with 1 (next minimal value). For all statistical analyses, a two-sided *p*-value < 0.05 was considered significant.

## Results

### Pure DCIS versus miCa: Tumour cells and TILs

Among the 131 analysed cases, 96 were classified as DCIS and 35 as miCa. As shown in Table 3, miCa were more frequently of high nuclear grade (*p* = 0.029, for grade 3), and contained more HER2+ and triple negative (TN) cases (*p* = 0.032). No other significant difference in tumour cell characteristics was demonstrated between DCIS and miCa.

**Table 3:** Pathobiological and TIL characteristics of pure DCIS (DCIS) and microinvasive carcinomas (miCa)

	DCIS (n = 96)	miCa (n = 35)	*p* value
Characteristic			
Lesion size (mm)	12 ± 16	14 ± 20	NS
Lesions ≥20 mm	21 (21.9%)	10 (28.6%)	NS
Architectural pattern			
comedo	26 (27.1)	14 (40.0%)	NS
solid	30 (31.3%)	16 (45.7%)	NS
cribriform	50 (52.1%)	15 (42.9%)	NS
micropapillary	31 (32.3%)	7 (20.0%)	NS
Presence of necrosis	61 (65.6%)	27 (77.1%)	NS
Presence of microcalcifications	71 (75.5%)	27 (77.1%)	NS
Nuclear grade			
low	14 (14.6%)	2 (6.3%)	NS
intermediate	45 (46.9%)	12 (34.3%)	NS
high	37 (38.5%)	21 (60.0%)	0.029
Mitotic index	3.7 ± 4.0	4.8 ± 4.4	NS
Ki67 index	4.8 ± 7.3	5.6 ± 6.4	NS
Molecular subtype			
luminal	67 (68.9%)	15 (42.9%)	
HER2	17 (17.7%)	11 (31.4%)	0.032*
triple negative	12 (12.5%)	9 (25.7%)	
TIL density (TIL-d)			
grade 0	15 (15.6%)	1 (2.9%)	NS (0.07)
grade 1	43 (44.8%)	16 (45.7%)	NS
grade 2	28 (29.2%)	9 (25.7%)	NS
grade 3	10 (10.4%)	9 (25.7%)	0.028
TIL phenotype			
cells CD8+	65 ± 91	150 ± 224	0.016
cells CD4+	106 ± 185	242 ± 320	0.001
cells FoxP3+	22 ± 40	44 ± 92	NS (0.08)
cells CD20+	134 ± 290	276 ± 504	NS
cells CD38+	28 ± 39	57 ± 73	0.024
T/B ratio	7.2 [5.3–9.2]	11.0 [5.2–16.8]	NS
CD8+/FoxP3+ ratio	9.4 [5.1–13.7]	11.1 [5.2–15.9]	NS

Legend: Values given for individual lymphocyte subpopulations are mean ± SD; values for the ratios of lymphocyte counts are means and 95% CI Abbreviations: *ER* oestrogen receptor, *PR* progesterone receptor, *NS* not significant * = *p* value for the difference in global distribution of molecular subtypes between DCIS and miCa

miCa contained significantly more cases with grade 3 TIL-d (≥50%) than the pure DCIS (*p* = 0.028). Although a statistically significant difference could not be demonstrated, miCa rarely contained minimal lymphocytic infiltration (grade 0 in only 1 case, 2.9%), whereas 15.6% of pure DCIS had that TIL-d grade (*p* = 0.07).

In terms of TIL composition, miCa contained significantly more CD8+, CD4+ and CD38+ cells (*p* = 0.016, *p* = 0.001 and *p* = 0.024, respectively) whereas no statistically significant difference between DCIS and miCa was observed in the number of FoxP3+ or CD20+ cells.

To compare pure DCIS and miCa by dominant immune response type at the tumour site, we derived a ratio (T/B ratio) between the cells representing cellular immunity (T-cells, CD8+ and CD4+) and the cells representing humoral immunity (B-cells, CD20+). Although the difference between pure and miCa did not reach statistical significance, the mean T/B ratio, as well as the upper limit of its range in miCa were higher than in the pure DCIS (Table 3). No significant difference either between pure DCIS and miCa was demonstrated in the ratio between the cytotoxic (CD8+) and regulatory (FoxP3+) T-cells.

### The “lymphocyte-rich” pure DCIS

After having observed that miCa very rarely display minimal lymphocytic infiltration, we wondered whether there are any common points between the pure DCIS with numerous stromal TILs and the miCa. We therefore separated the pure DCIS with TIL density of grade 2 and 3 from those with rare or absent TIL (grades 1 and 0) and designated the former as “lymphocyte-rich” DCIS (lyDCIS), whereas the lower TIL subcategory was named “lymphocyte-poor DCIS” (non-lyDCIS).

As shown in Table 4, when compared to the non-lyDCIS, the lyDCIS significantly differed in many pathobiological features: they were devoid of low nuclear grade cases (p = 0.001) but had significantly more high-grade ones (*p* = 0.0016) and contained more HER2+ lesions (*p* = 0.0059). In addition, the comedo architectural pattern and necrosis, as well as higher mitotic indices were more frequent in the lyDCIS than in the non-lyDCIS (p = 0.0016, *p* = 0.0065, *p* <  0.001, respectively).

**Table 4:** Pathobiological and TIL characteristics of lymphocyte-poor (non-lyDCIS) and lymphocyte-rich non-invasive DCIS (lyDCIS)

	non-lyDCIS (*n* = 58)	lyDCIS (*n* = 38)	*p* value
Characteristic			
Lesion size (mm)	11 ± 14	13 ± 19	NS
Lesions ≥20 mm	11 (18.6%)	11 (28.9%)	NS
Architectural pattern			
comedo	9 (15.5%)	17 (44.7%)	0.0016
solid	18 (31.0%)	12 (31.6%)	NS
cribriform	29 (50.0%)	21 (55.3%)	NS
micropapillary	23 (39.7%)	8 (21.1%)	NS
Presence of necrosis	32 (55.2%)	29 (82.9%)	0.0065
Presence of microcalcifications	44 (75.9%)	27 (75.0%)	NS
Nuclear grade			
low	14 (24.1%)	0 (0%)	0.001
intermediate	29 (50.0%)	16 (42.1%)	NS
high	15 (25.9%)	22 (57.9%)	0.0016
Mitotic index	2.4 ± 2.7	5.8 ± 4.7	< 0.001
Ki67 index	3.4 ± 4.4	6.9 ± 9.9	NS
Molecular subtype			
luminal	45 (77.6%)	22 (57.9%)	
HER2	4 (6.9%)	13 (34.2%)	0.0059*
triple negative	9 (15.5%)	3 (7.9%)	
TIL density (TIL-d)			
grade 0	15	0	NA
grade 1	43	0	NA
grade 2	0	28	NA
grade 3	0	10	NA
TIL phenotype			
cells CD8+	42 ± 64	102 ± 113	0.0002
cells CD4+	68 ± 138	165 ± 229	0.0018
cells FoxP3+	7 ± 15	47 ± 54	< 0.0001
cells CD20+	57 ± 123	253 ± 411	0.00013
cells CD38+	19 ± 33	41 ± 43	0.00051
T/B ratio	9.0 [6.2–11.7]	4.6 [2.4–11.7]	0.029
CD8+/FoxP3+ ratio	8.2 [5.1–11.3]	11.6 [0.8–22.4]	NS

Legend: Values given for individual lymphocyte subpopulations are mean ± SD; values for the ratios of lymphocyte counts are means and 95% CI. Abbreviations: *NA* not applicable, *NS* not significant, * = *p* value for the difference in global distribution of molecular subtypes between non-lyDCIS and lyDCIS

Interestingly, although all subtypes of TILs were more numerous in the lyDCIS than in the non-lyDCIS, the T/B ratio was significantly lower in the former, compared to the latter (*p* = 0.029).

No significant difference was demonstrated between the lyDCIS and the miCa either in pathobiology of the malignant cells or in the numbers of TIL subpopulations (Additional file 1). The T/B ratio was the only parameter which significantly differed between the lyDCIS and the miCa, being markedly higher in the miCa (4.6 [2.6–6.8] vs. 11.0 [5.2–16.8], lyDCIS vs miCa, respectively, *p* = 0.017).

### Cluster analysis

To better explore the relationship between non-lyDCIS, lyDCIS and miCa, we performed a cluster analysis which evaluated distribution of those 3 categories according to several pathobiological characteristics not related to TILs, reported in the literature to have prognostic significance in invasive or in situ BC: lesion size, nuclear grade, mitotic and Ki67 index, molecular subtype, presence of necrosis or microcalcifications.

**Fig. 2:**
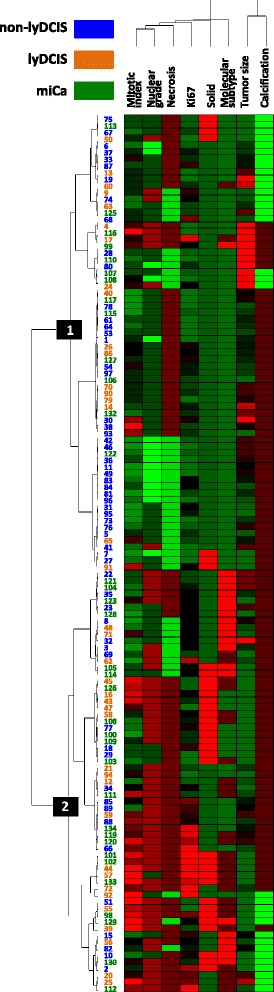
Cluster analysis of distribution of TIL-based subcategories according to the tumour cell pathobiological characteristics. Red colour represents higher values/presence of a parameter; green colour represents lower values/absence of a parameter. For molecular subtypes: green = luminal, brown = HER2+, red = triple negative. The numbers in blue, green and orange are IDs of the cases analysed

The entire cohort clustered in 2 main groups and that distribution was highly statistically significant (*p* = 0.017, Fig. 2). The non-lyDCIS clustered dominantly in group 1 (55.9% of cases, compared to 31.7% of cases found in the group 2) and that is the basis of the significant difference in case distribution between 2 clusters. The lyDCIS and the miCa clustered similarly in the 2 groups. Those findings confirmed pathobiological similarities between lyDCIS and miCa and their difference from non-lyDCIS.

### Prognostic value of TIL density and immunophenotype

No TIL characteristics was predictive of recurrence in this series. The only independent predictive factor of disease-free survival was solid architectural pattern of the tumors (*p* = 0.0023, Additional File 2).

## Discussion

Here we are reporting that assessment of stromal lymphocyte density and phenotype reveals two distinct subgroups of DCIS, one of which shows significant pathobiological similarities with miCa. In addition, we demonstrate that the TIL-rich pure DCIS are characterized by lower ratios of T- to B-TILs, either compared to the TIL-poor pure DCIS or to the miCa.

Several authors have already addressed the question of stromal lymphocytic infiltrate significance in DCIS with or without microinvasion [14–18]. In the seminal paper on DCIS immunogenicity, Black et al. suggested an association of cell-mediated immunity and the in situ phase of breast carcinogenesis [19]. To our best knowledge, our study is one of the largest which investigated TIL phenotype in DCIS, and the first which revealed differences in the ratio of T- to B-TILs between TIL-rich and TIL-poor DCIS as well as between pure DCIS and miCa.

It has already been demonstrated that high-grade pure DCIS and miCa lesions are frequently associated with rich lymphocytic infiltrate, amount of which is growing, in most cases, from normal breast tissue to the invasive cancer [15, 16, 20, 21]. Our results are in concordance with those reports. We observed minimal lymphocytic infiltrate only in one case of miCa and a significantly higher rate of grade 3 TIL-d in miCa than in the pure DCIS. No study yet compared TIL-d between pure DCIS and miCa by assessing it on H&E-stained breast biopsy sections. In a small series of DCIS (*n* = 27, only 3 miCa) Thompson et al. found that 78% of cases contained TILs occupying 5% or more of the stromal area [17]. Using a slightly different grading we also found that most DCIS have well observable TILs (lesions with ≥10% of TILs represent more than 80% of our cohort). We applied the recently published recommendations for TIL-d scoring in IBC [9], but arbitrarily established 4 grades, chosen by consensus of 4 authors (MB, MMD, FPL and NRR) on what could be easily and faithfully reproduced in the routine practice. Assessment of TIL-d on breast biopsies instead on surgical specimens is one of the limitations of this study, since TIL-d in a surgical specimen might not exactly be reflected by the corresponding biopsy. On the other side, to serve as a biomarker helpful for tailoring the initial DCIS treatment (e.g. surgery type), or for selection of pts. for pre-surgery or surgery-replacing immunotherapy trials, TIL-d must be assessable on breast biopsies.

There is still little knowledge about the content of DCIS stromal mononuclear infiltrate and its relationship to DCIS pathobiology. Lee et al. reported predominating T- and B-lymphocytes over relatively low number of macrophages in DCIS, whereas in the IBC T-lymphocytes and macrophages were the most frequent [14]. Thompson et al. observed that total T-lymphocyte population (CD3+) as well as the CD8+ and CD4+ subpopulations, followed by the CD20+ cells, were most numerous in all studied DCIS, whereas the FoxP3+ cells showed lower counts [17]. Campbell et al. reported higher counts of CD8+, CD4+, FoxP3+ and CD20+ cells in high grade pure DCIS, in comparison to the non-high grade cases [18]. Our results are comparable to those of Thompson et al. in terms of lower densities of the FoxP3+ cells in comparison to the other TILs. The TIL-rich DCIS in our cohort contained a high fraction (almost 60%) of high-grade DCIS and was richer in all investigated TIL phenotypes (CD8+, CD4+, FoxP3+, CD20+ and CD38+) than the TIL-poor DCIS subcategory, which contained much less high-grade lesions. This is comparable, in part, with the results of Campbell et al., however, our TIL-rich subcategory contained also DCIS cases of intermediate grade. The total absence of low grade lesions among the TIL-rich cases in our series suggests that low grade DCIS lesions are rarely associated with an intense immune reaction. In that line, as Campbell et al. evoked, high grade DCIS lesions have significantly different immune landscape than the rest of DCIS (more TILs, different TIL immunophenotype).

We discovered that the T/B ratio was reduced in the subgroup of pure DCIS with ≥30% TILs, in comparison either with the DCIS having less than 30% of TILs or with miCa. No previous study has investigated T/B ratio in breast carcinoma in situ. The lower T/B ratio might be provoked by an excess of B-lymphocytes. It has been demonstrated that HER2 elicits marked increase in interleukin-6 (IL-6) expression and secretion [22]. IL-6 acts on B-lymphocytes by increasing their immunoglobulin production [23]. In addition, B-lymphocytes secrete IL-6 themselves [23], forming an autocrine loop which might be especially productive in the HER2+ preinvasive and invasive breast lesions. Indeed, our TIL-rich DCIS group, showing higher B-cell counts than the TIL-poor DCIS category, contained more HER2+ cases, so that is likely the strongest explanation of a relative excess of B-cells in the TIL-rich subgroup. On the other side, IL-6 is secreted also by adipose cells and may induce B-cell proliferation in the HER2-negative breast cancer lesions [24]. Thus, lower T/B ratio of the TIL-rich DCIS may also reflect the presence of microenvironment-induced chronic inflammation, demonstrated to constitute a milieu that stimulates breast carcinogenesis [25, 26]. The increased proportion of B-lymphocytes in the TIL-rich DCIS might indicate development of a pro-invasive milieu which will allow for progression toward the invasive disease. This hypothesis, however, should be verified in further studies.

Microinvasion is considered as the earliest step in the development of IBC. Once penetrated the basal lamina, DCIS cells can induce activation of the cytotoxic immune response, especially in case of HER2+ and TN lesions. HER2+ and TN malignant breast cells are considered highly immunogenic due to their frequent exposure of cancer-associated antigens [27–31]. In our cohort, predominance of CD8+ and CD4+ cells over B-cells is the strongest in miCa and likely reflects the situation in which the cellular immune response has started developing against invading malignant cells. The miCa subgroup contained more HER2+ and TN lesions than the rest of the cohort, so that is likely one of the strongest reasons for higher counts of the effector T-lymphocytes in the miCa.

As the adaptive immune response to cancer is characterized by progressive development of TILs with a role to reduce the anti-tumour action of CD8+ cells, we were interested whether increase in FoxP3+ T-cells, the major counter-actors of CD8+ T-cells [32, 33], will follow the increase in CD8+ TILs. We did observe increased numbers of FoxP3+ TILs in the TIL-rich DCIS compared to the TIL-poor DCIS. Lal et al. have reported increasing of FoxP3+ lymphocyte number along the malignant progression from normal breast tissue to IBC [16]. Our finding of increasing FoxP3+ cell counts but no significant changes in the CD8+/FoxP3+ ratio from non-lyDCIS to lyDCIS and miCa might indicate that the control of malignant cell population by the cellular immune response is still operational and not inhibited by immunoediting [34]. To confirm this hypothesis, it would be worth investigating whether the reduced ratios of CD8+ to FoxP3+ TILs are present in breast cancer lesions with more extended invasion (> 1 mm).

By cluster analysis of several important pathobiological characteristics we demonstrated that miCa and the TIL-rich pure DCIS are closely related and different from the DCIS with low or absent TILs. The similarity between miDCIS and lyDCIS could be explained by high rate of HER2+ cases in both categories (34–35%), markedly higher than in the non-lyDCIS (only 6.8%). HER2+ subtype is frequently found in miCa [35, 36] and the association with HER2 positivity and rich TILs in DCIS has been reported [17, 18]. However, in our cohort, the HER2+ cases represented only slightly above one third of lyDCIS or miCa, suggesting that the HER2- in situ lesions could also induce development of rich lymphocytic infiltrate. The relatively frequent TN lesions (around one quart of the cases) could also be one of the reasons for denser TILs in the miCa compared to the rest of the cohort, however, interestingly, the TIL-rich pure DCIS category had less TN cases than the TIL-poor group. To better determine whether the similarities between TIL-rich DCIS and miCa are caused by factors unrelated to HER2+ or TN status we are currently investigating the relationship between TIL density and tumour cell pathobiological features in a larger series of luminal/HER2-negative pure DCIS and miCa.

We could not demonstrate an increased risk for recurrence of the miCa or the lyDCIS, because of the low number of recurrences in this series. Several authors have reported a very good prognosis of miCa [37–39], whereas others have stressed the clinical problem of local recurrences which would need prevention by large surgical excisions and adjuvant radiotherapy [40, 41]. Recently a fatal systemic progression of HER2+ miCa has been reported [42] implying that search for microinvasion foci in a DCIS lesion, especially of the HER2+ subtype, should be performed as carefully as possible. Our finding of significant similarities between lyDCIS and miDCIS suggest that denser TILs (≥30%) in a DCIS lesion should be evaluated, in larger series, as an indicator of invasion.

This study has limitations. First, use of TMAs for analysis of cellular densities cannot always ensure equal size of the areas on which cell counts are determined. However, this obstacle is largely overridden when ratios between the cell counts are used, as the counts are obtained on the same surface. For that reason, the T/B ratio used in this study likely was not influenced by the errors due to non-uniform size of the area within which TILs were counted.

Another limitation is still a relatively small cohort size, which could not allow for more details in the statistical analysis, especially with regards to the above evoked relationship between molecular subtypes and TIL density or immunophenotype.

## Conclusion

In conclusion, this study reveals two new subgroups of breast DCIS, which differ in amount and phenotype of TILs and in several tumour cell characteristics. However, this separation of pure DCIS into two subcategories, based on TIL density level, does not seem to be a mere reflect of the frequency of molecular subtypes. The subcategory of pure DCIS with rich TILs has a lower T/B ratio, which importance for invasion risk is worth investigating in larger studies. Analysis of TIL phenotype may reveal DCIS subtypes with low risk of progression (like DCIS with less than 30% TILs) for which the intensity of adjuvant treatment could be reduced. On the other side, if the association between relative excess of B-TILs and DCIS invasion is confirmed, preventive approaches based on B-cell immunity modulation could be envisioned.

## Additional files

Additional file 1: Pathobiological and TIL characteristics of lymphocyte-rich (lyDCIS) and microinvasive carcinomas (miCa) corresponds to the title. (DOCX 15 kb)

Additional file 2: Solid architectural pattern of DCIS lesions is predictive of shorter disease-free survival. Survival curves of patients having DCIS with solid and non-solid architectural pattern. (PPTX 40 kb)

## Abbreviations

BC: Breast cancer
DCIS: Ductal carcinoma in situ
ER: Oestrogen receptor
H&E: Haematoxylin & eosin
HER2: Human Epidermal growth factor Receptor 2
IBC: Invasive breast cancer
IHC: Immunohistochemistry
IL-6: Interleukin 6
ISH: In situ hybridization
lyDCIS: Lymphocyte-rich ductal carcinoma in situ
miCa: Microinvasive carcinoma
non-lyDCIS: Lymphocyte-poor ductal carcinoma in situ
PR: Progesterone receptor
pts.: Patients
TIL-d: Density of tumour-infiltrating lymphocytes
TILs: Tumour-infiltrating lymphocytes
TMA: Tissue microarray
TN: Triple negative

## References

[1] Siegel RL, Miller KD, Jemal A (2016). Cancer statistics, 2016. CA Cancer J Clin.

[2] Hoffman AW, Ibarra-Drendall C, Espina V, Liotta L, Seewaldt V. Ductal carcinoma in situ: challenges, opportunities, and uncharted waters. Am Soc Clin Oncol Educ Book. 2012:40–4. 10.14694/EdBook_AM.2012.32.228 24451705

[3] Morrow M, Schnitt SJ, Norton L (2015). Current management of lesions associated with an increased risk of breast cancer. Nat Rev Clin Oncol.

[4] Pang JM, Gorringe KL, Fox SB (2016). Ductal carcinoma in situ - update on risk assessment and management. Histopathology.

[5] Spira A, Disis ML, Schiller JT, Vilar E, Rebbeck TR, Bejar R, Ideker T, Arts J, Yurgelun MB, Mesirov JP (2016). Leveraging premalignant biology for immune-based cancer prevention. Proc Natl Acad Sci U S A.

[6] Savas P, Salgado R, Denkert C, Sotiriou C, Darcy PK, Smyth MJ, Loi S (2016). Clinical relevance of host immunity in breast cancer: from TILs to the clinic. Nat Rev Clin Oncol.

[7] Pusztai L, Karn T, Safonov A, Abu-Khalaf MM, Bianchini G (2016). New strategies in breast cancer: immunotherapy. Clin Cancer Res.

[8] Lakhani SR, Ellis IO, Schnitt SJ, Tan PH, Van de Vijver MJ (2012). WHO classification of Tumours of the breast.

[9] Salgado R, Denkert C, Demaria S, Sirtaine N, Klauschen F, Pruneri G, Wienert S, Van den Eynden G, Baehner FL, Penault-Llorca F (2015). The evaluation of tumor-infiltrating lymphocytes (TILs) in breast cancer: recommendations by an international TILs working group 2014. Ann Oncol.

[10] Allred DC, Harvey JM, Berardo M, Clark GM (1998). Prognostic and predictive factors in breast cancer by immunohistochemical analysis. Mod Pathol.

[11] Wolff AC, Hammond ME, Hicks DG, Dowsett M, McShane LM, Allison KH, Allred DC, Bartlett JM, Bilous M, Fitzgibbons P (2014). Recommendations for human epidermal growth factor receptor 2 testing in breast cancer: American Society of Clinical Oncology/College of American Pathologists clinical practice guideline update. Arch Pathol Lab Med.

[12] Goldhirsch A, Wood WC, Coates AS, Gelber RD, Thurlimann B, Senn HJ, Panel M (2011). Strategies for subtypes--dealing with the diversity of breast cancer: highlights of the St. Gallen international expert consensus on the primary therapy of early breast cancer. Ann Oncol 2011.

[13] Kwiatkowski F, Girard M, Hacene K, Berlie J (2000). Sem: a suitable statistical software adaptated for research in oncology. Bull Cancer.

[14] Lee AH, Happerfield LC, Bobrow LG, Millis RR (1997). Angiogenesis and inflammation in ductal carcinoma in situ of the breast. J Pathol.

[15] Hussein MR, Hassan HI (2006). Analysis of the mononuclear inflammatory cell infiltrate in the normal breast, benign proliferative breast disease, in situ and infiltrating ductal breast carcinomas: preliminary observations. J Clin Pathol.

[16] Lal A, Chan L, Devries S, Chin K, Scott GK, Benz CC, Chen YY, Waldman FM, Hwang ES (2013). FOXP3-positive regulatory T lymphocytes and epithelial FOXP3 expression in synchronous normal, ductal carcinoma in situ, and invasive cancer of the breast. Breast Cancer Res Treat.

[17] Thompson E, Taube JM, Elwood H, Sharma R, Meeker A, Warzecha HN, Argani P, Cimino-Mathews A, Emens LA (2016). The immune microenvironment of breast ductal carcinoma in situ. Mod Pathol.

[18] Campbell MJ, Baehner F, O'Meara T, Ojukwu E, Han B, Mukhtar R, Tandon V, Endicott M, Zhu Z, Wong J, et al. Characterizing the immune microenvironment in high-risk ductal carcinoma in situ of the breast. Breast Cancer Res Treat. 2016; 10.1007/s10549-016-4036-0 PMC5225037 27785654

[19] Black MM, Zachrau RE, Hankey BF, Feuer EJ (1996). Prognostic significance of in situ carcinoma associated with invasive breast carcinoma. A natural experiment in cancer immunology?. Cancer.

[20] Ben-Hur H, Cohen O, Schneider D, Gurevich P, Halperin R, Bala U, Mozes M, Zusman I (2002). The role of lymphocytes and macrophages in human breast tumorigenesis: an immunohistochemical and morphometric study. Anticancer Res.

[21] Morita M, Yamaguchi R, Tanaka M, Tse GM, Yamaguchi M, Otsuka H, Kanomata N, Minami S, Eguchi S, Yano H. Two progressive pathways of microinvasive carcinoma: low-grade luminal pathway and high-grade HER2 pathway based on high tumour-infiltrating lymphocytes. J Clin Pathol. 2016; 10.1136/jclinpath-2015-203506 27030304

[22] Hartman ZC, Yang XY, Glass O, Lei G, Osada T, Dave SS, Morse MA, Clay TM, Lyerly HK (2011). HER2 overexpression elicits a proinflammatory IL-6 autocrine signaling loop that is critical for tumorigenesis. Cancer Res.

[23] Vazquez MI, Catalan-Dibene J, Zlotnik A (2015). B cells responses and cytokine production are regulated by their immune microenvironment. Cytokine.

[24] Walter M, Liang S, Ghosh S, Hornsby PJ, Li R (2009). Interleukin 6 secreted from adipose stromal cells promotes migration and invasion of breast cancer cells. Oncogene.

[25] Iyengar NM, Hudis CA, Dannenberg AJ. Obesity and inflammation: new insights into breast cancer development and progression. Am Soc Clin Oncol Educ Book. 2013:46–51. 10.1200/EdBook_AM.2013.33.46 PMC3897299 23714453

[26] Jiang X, Shapiro DJ (2014). The immune system and inflammation in breast cancer. Mol Cell Endocrinol.

[27] Coronella JA, Telleman P, Kingsbury GA, Truong TD, Hays S, Junghans RP (2001). Evidence for an antigen-driven humoral immune response in medullary ductal breast cancer. Cancer Res.

[28] Chen YT, Ross DS, Chiu R, Zhou XK, Chen YY, Lee P, Hoda SA, Simpson AJ, Old LJ, Caballero O (2011). Multiple cancer/testis antigens are preferentially expressed in hormone-receptor negative and high-grade breast cancers. PLoS One.

[29] Ademuyiwa FO, Bshara W, Attwood K, Morrison C, Edge SB, Karpf AR, James SA, Ambrosone CB, O'Connor TL, Levine EG (2012). NY-ESO-1 cancer testis antigen demonstrates high immunogenicity in triple negative breast cancer. PLoS One.

[30] Wang H, Sang M, Geng C, Liu F, Gu L, Shan B (2016). MAGE-A is frequently expressed in triple negative breast cancer and associated with epithelial-mesenchymal transition. Neoplasma.

[31] Luen S, Virassamy B, Savas P, Salgado R, Loi S (2016). The genomic landscape of breast cancer and its interaction with host immunity. Breast.

[32] Fontenot JD, Gavin MA, Rudensky AY (2003). Foxp3 programs the development and function of CD4+CD25+ regulatory T cells. Nat Immunol.

[33] Facciabene A, Motz GT, Coukos G (2012). T-regulatory cells: key players in tumor immune escape and angiogenesis. Cancer Res.

[34] Mittal D, Gubin MM, Schreiber RD, Smyth MJ (2014). New insights into cancer immunoediting and its three component phases--elimination, equilibrium and escape. Curr Opin Immunol.

[35] Yang M, Moriya T, Oguma M, De La Cruz C, Endoh M, Ishida T, Hirakawa H, Orita Y, Ohuchi N, Sasano H (2003). Microinvasive ductal carcinoma (T1mic) of the breast. The clinicopathological profile and immunohistochemical features of 28 cases. Pathol Int.

[36] Mori M, Tsugawa K, Yamauchi H, Yagata H, Suzuki K, Ohde S, Soejima K, Nakamura S (2013). Pathological assessment of microinvasive carcinoma of the breast. Breast Cancer.

[37] Silver SA, Tavassoli FA (1998). Mammary ductal carcinoma in situ with microinvasion. Cancer.

[38] Margalit DN, Sreedhara M, Chen YH, Catalano PJ, Nguyen PL, Golshan M, Overmoyer BA, Harris JR, Brock JE (2013). Microinvasive breast cancer: ER, PR, and HER-2/neu status and clinical outcomes after breast-conserving therapy or mastectomy. Ann Surg Oncol.

[39] Wang L, Zhang W, Lyu S, Liu X, Zhang T, Liu S, Qin Y, Tian X, Niu Y (2015). Clinicopathologic characteristics and molecular subtypes of microinvasive carcinoma of the breast. Tumour Biol.

[40] Solin LJ, Fowble BL, Yeh IT, Kowalyshyn MJ, Schultz DJ, Weiss MC, Goodman RL (1992). Microinvasive ductal carcinoma of the breast treated with breast-conserving surgery and definitive irradiation. Int J Radiat Oncol Biol Phys.

[41] de Mascarel I, MacGrogan G, Mathoulin-Pelissier S, Soubeyran I, Picot V, Coindre JM (2002). Breast ductal carcinoma in situ with microinvasion: a definition supported by a long-term study of 1248 serially sectioned ductal carcinomas. Cancer.

[42] Kuhar CG, Matos E (2014). Human epidermal growth factor receptor 2-positive microinvasive breast carcinoma with a highly aggressive course: a case report. BMC Res Notes.

