# Supplementary material for: Analysis of tumour-infiltrating lymphocytes reveals two new biologically different subgroups of breast ductal carcinoma in situ
Source: BMC Cancer. 2018 Feb 3;18:129. doi: 10.1186/s12885-018-4013-6 (PMC5797400; doi:10.1186/s12885-018-4013-6)
Supplement: Supplementary file 1 — Pathobiological and TIL characteristics of lymphocyte-rich (lyDCIS) and microinvasive carcinomas (miCa) corresponds to the title. (DOCX 15 kb) [file 12885_2018_4013_MOESM1_ESM.docx]

Additional file 1: Pathobiological and TIL characteristics of lymphocyte-rich (lyDCIS) and microinvasive carcinomas (miCa)

|  | lyDCIS  (n=38) | miCa  (n=35) | p value |
| --- | --- | --- | --- |
| Characteristic |  |  |  |
|  |  |  |  |
| Lesion size (mm) | 13±19 | 14±20 | NS |
| Lesions ≥ 20mm | 11 (28.9%) | 10 (28.6%) | NS |
|  |  |  |  |
| Architectural pattern |  |  |  |
| comedo | 17 (44.7%) | 14 (40.0%) | NS |
| solid | 12 (31.6%) | 16 (45.7%) | NS |
| cribriform | 21 (55.3%) | 15 (42.9%) | NS |
| micropapillary | 8 (21.1%) | 7 (20.0%) | NS |
|  |  |  |  |
| Presence of necrosis | 29 (82.9%) | 27 (77.1%) | NS |
| Presence of microcalcifications | 27 (75.0%) | 27 (77.1%) | NS |
|  |  |  |  |
| Nuclear grade |  |  |  |
| low | 0 (0%) | 2 (6.3%) | NS |
| intermediate | 16 (42.1%) | 12 (34.3%) | NS |
| high | 22 (57.9%) | 21 (60.0%) | NS |
|  |  |  |  |
| Mitotic index | 5.8±4.7 | 4.8±4.4 | NS |
| Ki67 index | 6.9±9.9 | 5.6±6.4 | NS |
|  |  |  |  |
| Molecular subtype |  |  |  |
| luminal | 22 (57.9%) | 15 (42.9%) | NS |
| HER2 | 13 (34.2%) | 11 (31.4%) | NS |
| triple negative | 3 (7.9%) | 9 (25.7%) | NS |
|  |  |  |  |
| TIL density (TIL-d) |  |  |  |
| grade 0 | 0 | 1 (2.9%) | NA |
| grade 1 | 0 | 16 (45.7%) | NA |
| grade 2 | 28 *(73.7%)* | 9 (25.7%) *(50.0%)* | NA |
| grade 3 | 10 *(26.3%)* | 9 (25.7%) *(50.0%)* | NA |
|  |  |  |  |
| TIL phenotype |  |  |  |
| cells CD8+ | 102±112 | 150±224 | NS |
| cells CD4+ | 165±229 | 242±320 | NS |
| cells FoxP3+ | 47±54 | 44±92 | NS |
| cells CD20+ | 253±411 | 276±504 | NS |
| cells CD38+ | 41±43 | 57±73 | NS |
| T/B ratio | 4.6 [2.6-6.8] | 11.0 [5.2-16.8] | 0.017 |
| CD8+/FoxP3+ ratio | 11.6 [0.8-22.4] | 11.1 [5.2-15.9] | NS |

Legend: Values given for individual lymphocyte subpopulations are mean±SD; values for the ratios of lymphocyte counts are means and 95% CI. Abbreviations: NS = not significant, NA = not applicable. In *italic*: percentages of each TIL-d grade within the grade 2+3 group.
