# Supplementary figures and images for: Analysis of tumour-infiltrating lymphocytes reveals two new biologically different subgroups of breast ductal carcinoma in situ
Source: BMC Cancer. 2018 Feb 3;18:129. doi: 10.1186/s12885-018-4013-6 (PMC5797400; doi:10.1186/s12885-018-4013-6)

## Slide 1
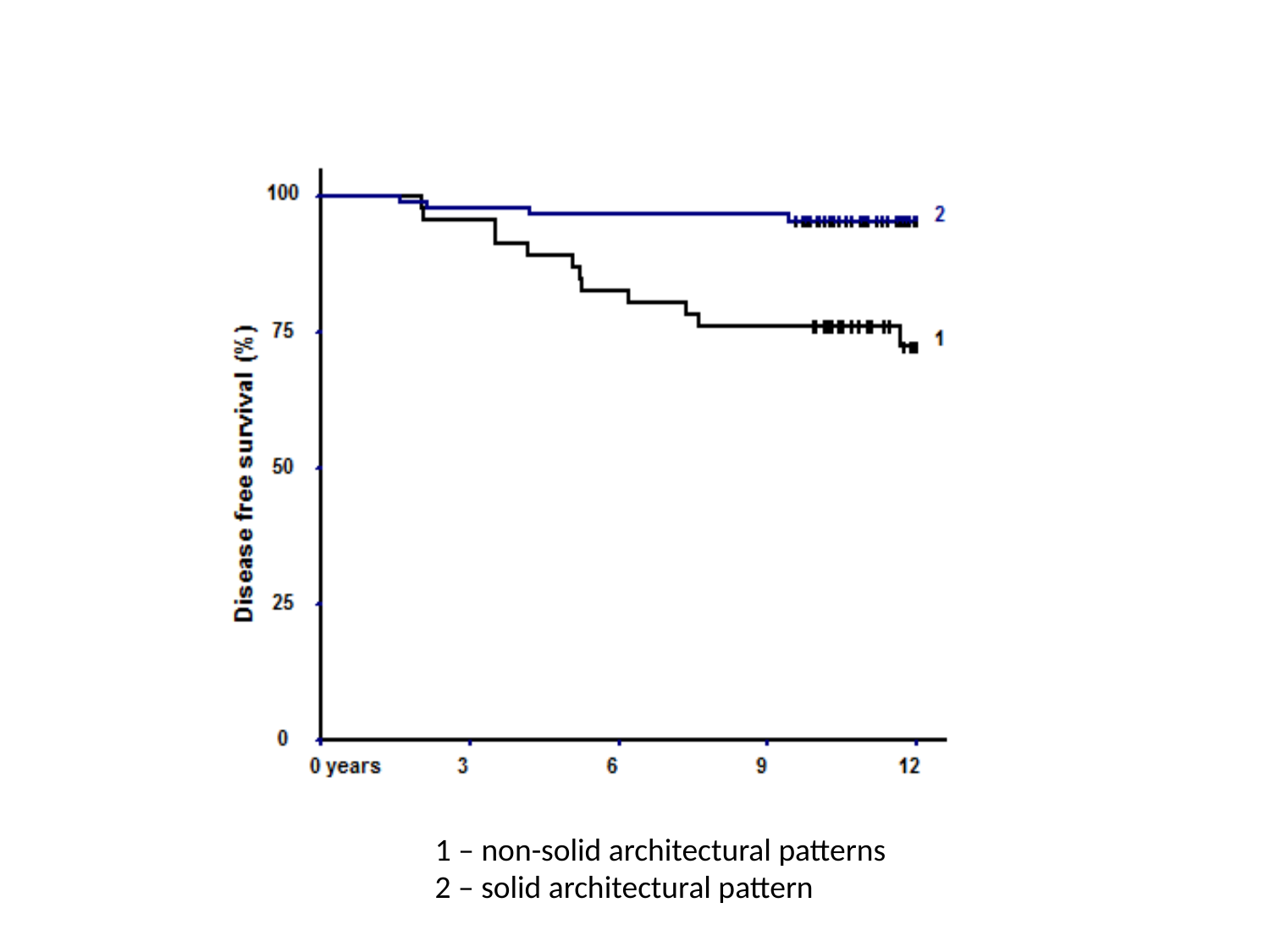

1 – non-solid architectural patterns
2 – solid architectural pattern

Supplement: Supplementary file 2 — Solid architectural pattern of DCIS lesions is predictive of shorter disease-free survival. Survival curves of patients having DCIS with solid and non-solid architectural pattern. (PPTX 40 kb) [file 12885_2018_4013_MOESM2_ESM.pptx]
